# Adaptation of *Anopheles* Vectors to Anthropogenic Malaria-Associated Rubber Plantations and Indoor Residual Spraying: Establishing Population Dynamics and Insecticide Susceptibility

**DOI:** 10.1155/2018/9853409

**Published:** 2018-06-21

**Authors:** Suntorn Pimnon, Adisak Bhumiratana

**Affiliations:** ^1^Faculty of Public Health, Bangkokthonburi University, Bangkok 10170, Thailand; ^2^Department of Parasitology and Entomology, Faculty of Public Health, Mahidol University, Bangkok 10400, Thailand; ^3^Center of Ecohealth Education and Research (CEER), Faculty of Public Health, Thammasat University, Rangsit Campus, Pathumthani 12121, Thailand

## Abstract

Anthropogenic activity such as the establishment of *Anopheles*-infested rubber plantations can influence local malaria transmission dynamics to which the population dynamics and insecticide susceptibility of local *Anopheles* vectors are related. Using human landing catch collections at a house protected by indoor residual spraying (IRS), the periodic assessment of species composition, abundance, and blood-feeding behaviors was done in pre-IRS, during IRS, and post-IRS at 3, 6, and 12 months in a malaria-associated rubber plantation (MRP) ecotope of the Bo Rai district, Trat Province, Thailand, after malaria outbreak occurred. The study MRP ecotope elicited the population ratio (*p*_*i*_) of *Anopheles* vectors: *An. campestris* (0.747), *An. dirus* (0.168), *An. minimus* (0.037), *An. barbirostris* (0.027), and *An. pseudowillmori* (0.002), and of *An. jamesii* nonvector (0.019). Among these, two predominant *An. campestris* and *An. dirus* night-biters were then used in the susceptibility test against 0.05% deltamethrin (DEL) and 0.09% bifenthrin (BT) insecticides currently used in IRS. *An. campestris*, a suspected vector of *Plasmodium vivax*, had a tendency to appear throughout the study and behaved both exophagy and endophagy. It was highly susceptible to BT, showing 95.0% mortality (95% CI, 79.1–100) while decreasing sensitivity of 87.2% (95% CI, 78.4–95.9) to DEL. *An. dirus*, a primary vector of *Plasmodium falciparum*, had a tendency to feed outdoors rather than indoors. Significant differences in the abundance (mean density and human landing rate) were observed at pre-IRS (*P* < 0.001  and  *P*=0.046), and similarly, during IRS (*P*=0.001  and  *P*=0.037). It was highly susceptible to DEL and BT, showing 100% mortality rate. Evidently, the study MRP ecotope contributed receptive environment to favor the abundant local *Anopheles* vectors and their outdoor biting preference. This can pose the risk for residual malaria parasite transmission in *Anopheles* vectors even though the house is protected by IRS.

## 1. Introduction

Land-use and land-cover change in the Earth's ecosystems reflects human interactions with the natural environment and increasingly, has been addressed as a primary source of global environmental change [1–3]. The anthropogenic factors that contribute significantly to land-use change become essential for studying human-environment interactions. Particularly, deforestation and other anthropogenic landscapes influence biophysical environmental conditions of the land system, habitat change, and invasive species [4].

**Figure 1:**
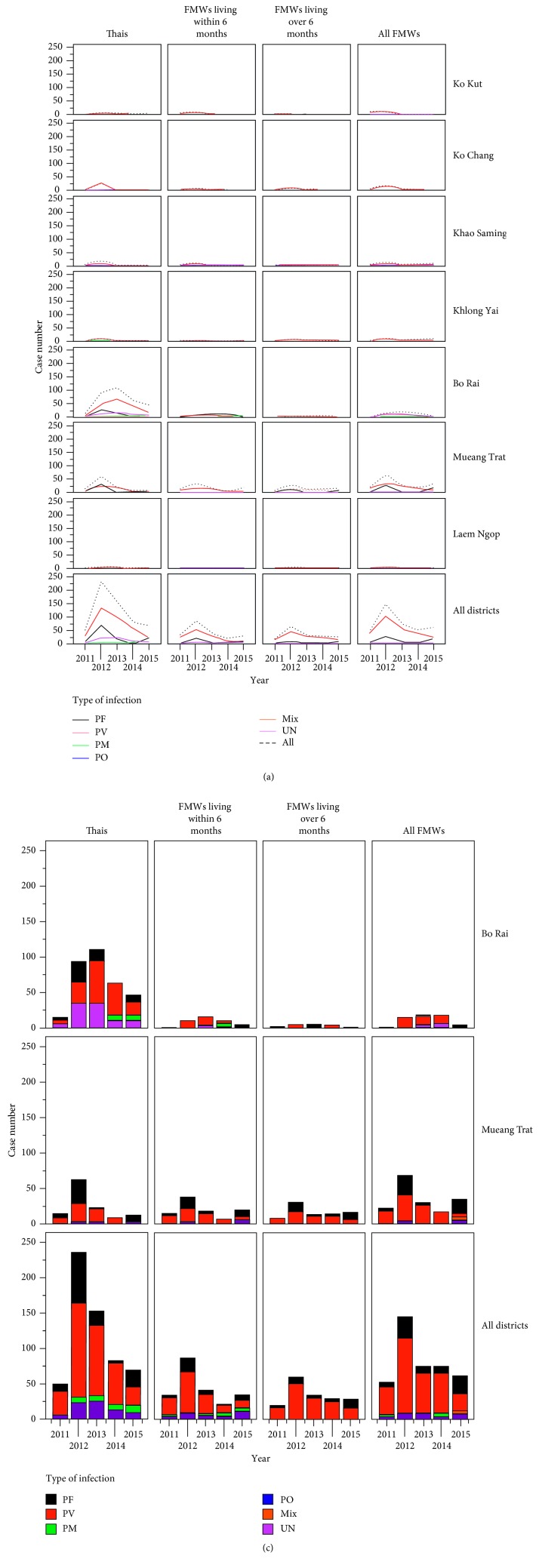
Malaria incidence for Trat, 2011–2015. (a) A trend of malaria incidence showing annually reported malarial cases with the types of *Plasmodium* infection between 2011 and 2015 at the district level of Trat Province. The local Thai populations were more likely to be affected by malaria than foreign migrant workers. Dashed line is shown for overall malarial case numbers. (b) Annual malaria incidence mostly reported by two following districts, Bo Rai and Mueang Trat, as compared to overall malarial case numbers. FMWs: foreign migrant workers; PF: *Plasmodium falciparum*; PV: *Plasmodium vivax*; PM: *Plasmodium malariae*; PO: *Plasmodium ovale*; UN: unknown malarial parasites. Data were modified from the Bureau of Vector-Borne Diseases, Department of Disease Control, Ministry of Public Health, Thailand.

Anthropogenic activity such as the establishment or expansion of rubber plantations [5, 6] can influence the agricultural land use changes and agrienvironmental climatic conditions that can cause human impacts of malaria [5–8]. For instance, over two decades of large-scale forestry cultures including rubber plantations in Thailand influence many receptive land areas of rubber plantations that potentially established the emergence of malaria outbreaks [5–7]. Trat Province located in the eastern region of Thailand has known endemic malaria transmission confined to Thai-Cambodia border settings and land areas of rubber plantations. Overall, the province has remarkably shown a trend of dramatic decreases in malaria case numbers annually reported by the districts (Figure 1). However, Bo Rai district showed ongoing transmission with a 2012-2013 upward trend. Most affected persons including local Thais rather than other foreign migrant workers were involved in routine rubber plantation practices including rubber tapping during the night time. This key district has established traditional rubber plantations by replacing oil palm plantations and mixed fruit orchards since the early 1990s. Land transformations to rubber plantations have resulted in local malaria transmission dynamics whether emerged or reemerged at high levels of malaria incidence, particularly in the South, East, and Northeast of Thailand [5].

The malaria-associated rubber plantation (MRP) ecotope is the unique landscape structure [5, 6], that is, both topographically shaped by rubber plantation polygons in which rubber trees are either monocultured or mixed with other perennial trees or orchards and geographically associated with the infestation or reinfestation of *Anopheles* vectors [9]. The potential MRP ecotope to which the shaded environment of rubber plantations is related can create microclimatic conditions favoring the availability of *Anopheles* larval habitats and the abundance of *Anopheles* vectors [5, 9–12]. If expected to accompany highly dynamically micro-agrienvironmental and climatic conditions, diverse MRP ecotopes are likely to become ecoepidemiological complex settings that can potentially drive the dynamics of malaria vector populations and human-vector interactions. If more adversely affected under these circumstances, the dynamics of vulnerability to malaria transmission can be the results of the increased susceptibility of vulnerable persons exposed to local *Anopheles* vectors [5, 7, 9] that have the ability to adapt to changing environmental conditions from natural forest habitats to rubber plantations as cultivated forest habitats.

The MRP ecotopes pertaining to human-induced land-use changes have known significant sites for surveillance of *Anopheles* vector populations and malaria transmission [5, 6, 8, 9, 13]. *Anopheles* vector surveillance focuses on species composition, abundance, blood-feeding behaviors, and on insecticide susceptibility of local *Anopheles* vectors adapted to biophysical conditions of receptive rubber plantations. Due to the outdoor exposure to frequent bites of local *Anopheles* vectors, residual malaria parasite transmission is needed to monitor the *Anopheles* vector infections even though the houses are protected by the implementation of core interventions of vector control including indoor residual spraying (IRS), insecticide-treated nets (ITNs), and long-lasting insecticidal nets (LLINs) [5, 13].

Thus, the ultimate goal of this study was to assess the adaptation of local *Anopheles* vectors to local environment of an emerged MRP ecotope site of the Bo Rai district, Trat Province, where the IRS using the pyrethroids had been implemented to interrupt the local malaria transmission. First, we analyzed the population dynamics of locally adapted *Anopheles* vectors, based radically on variations of species composition, abundance, and blood-feeding behaviors of *Anopheles* vectors that were observed in pre-IRS, during IRS, and post-IRS. Lastly, we tested the susceptibility of wild-caught population samples of some potential *Anopheles* vectors against the pyrethroids that were currently used in vector control.

## 2. Methods

### 2.1. Study Plan

#### 2.1.1. Background Information of the Emerged MRP Ecotope

**Figure 2:**
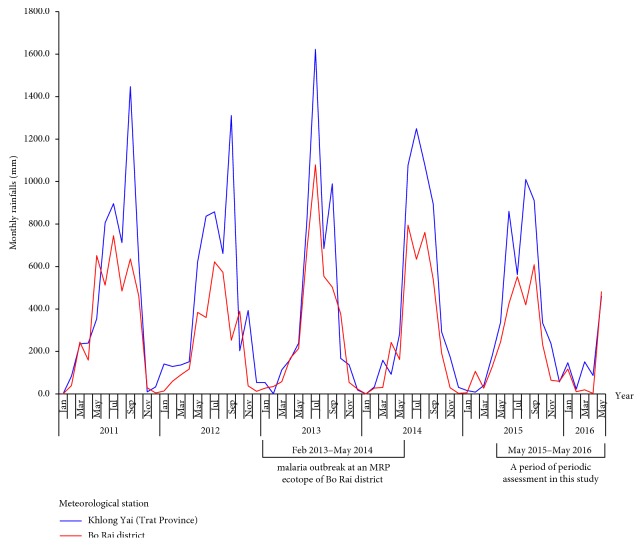
Rainfall patterns. Monthly rainfall data are shown for the period, from January 2011 to May 2016, for Trat province and Bo Rai district. Also, monthly rainfall patterns are shown during which the malaria outbreak occurred at Khlong Khak MRP ecotope of the Bo Rai district, and assessment of *Anopheles* species composition, abundance, and blood-feeding behaviors was carried out.

**Figure 3:**
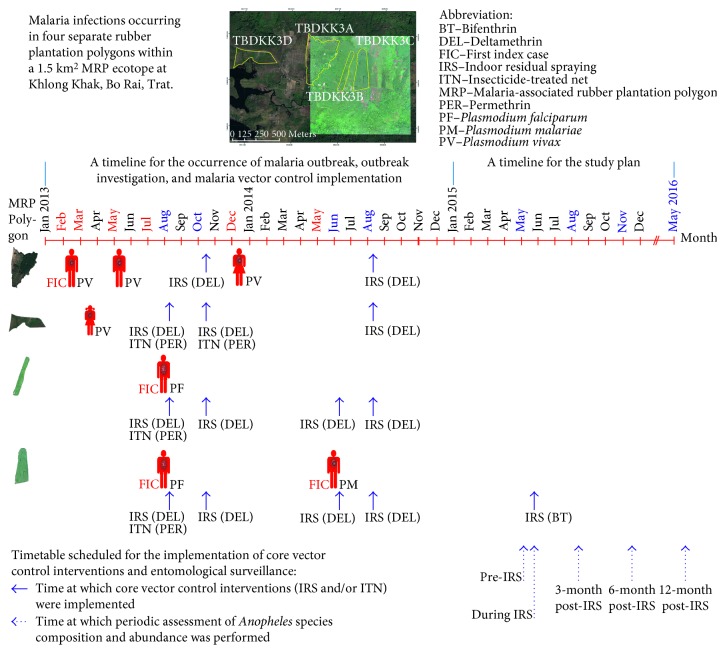
A schematic presentation of a timeline of the occurrence of malaria outbreak, outbreak investigation, malaria vector control implementation, and study plan. The *Plasmodium* infections occurring in four separate rubber plantation polygons in which the polygon TBDKK3C was selected as the study site after the outbreak was locally arrested by the implementation of core interventions of the vector control using IRS and ITNs.

The study MRP ecotope that established the emergence of malaria outbreak between 2013 and 2014 was located in Khlong Khak village, Bo Rai district, Trat Province, close to the Thai-Cambodia border [6]. The outbreak was likely to relate high rainfall states to malaria risk (Figure 2). All households residing in four separate rubber plantation polygons of this study MRP ecotope were involved in rubber plantation practices. In this outbreak, the vector control measures using IRS and ITNs were household-level implemented in the absence of vector surveillance (Figure 3). Between August and October 2013, the Vector-Borne Disease Control Unit (VBDU) 6.4.4 Bo Rai, belonging to the Vector-Borne Disease Control Center (VBDC) 6.4, had implemented the IRS using deltamethrin (DEL) insecticide as the first-line malaria vector control to interrupt local malaria transmission. But there existed two more rubber tappers who developed newly *P. vivax* infection in December 2013, and later, newly *P. malariae* infection in June 2014. Finally, it was locally arrested by the IRS and ITNs. The DEL had been used until August 2014. The bifenthrin (BT) insecticide was recommended for use as the second-line malaria vector control in 2015.

The outbreak investigation and response were implemented according to the guidelines of the National Malaria Control Program (NMCP) by the VBDC 6.4 Trat as the first responder, belonging to the Office of Disease Prevention and Control (ODPC) 6 Chonburi. However, the outbreak investigation lacked baseline information of species composition, abundance, and blood-feeding behaviors of local *Anopheles* vectors. Neither the fluctuations of the *Anopheles* vector abundances that relate to changes in monthly rainfall patterns nor the variations of human blood-feeding behaviors and insecticide susceptibility of local *Anopheles* vectors in this study MRP ecotope have been documented.

#### 2.1.2. Study Design

The study was followed by the recent 2013-2014 malaria outbreak at Khlong Khak MRP ecotope as mentioned earlier. The study protocols were designed to better understand the vulnerability in how local *Anopheles* vectors are adapted to local environment (or certain biophysical conditions) of this emerged MRP ecotope and how they respond to the IRS using the pyrethroids (or certain chemical constraints) currently used in the NMCP. Prior to the study, *Anopheles* larval surveys were performed between January and May 2015 in order to determine the extent to which this MRP ecotope had the potential density of *Anopheles* larval breeding sites. This permitted selection of a house whose two rubber tappers developed the infections with *P. falciparum* and *P. malariae* within two consecutive years, as described in detail later.

In this study, a selected house was treated by the IRS using BT. This permitted assessment of the effects of IRS on the dynamics of *Anopheles* vector populations by periodic assessment of species composition, abundance, and blood-feeding behaviors of local *Anopheles* vectors; all of which were collected by human landing catches, starting from May 2015 to May 2016 (Figures 2 and 3) as described in detail later. Comparisons were performed on the mean densities or human landing rates of night-biting *Anopheles* female adult populations derived from indoor and outdoor collections in pre-IRS, during IRS, and post-IRS. At the time of the mosquito collection, the study tested the susceptibility of pooled population samples of some potential *Anopheles* vectors against the pyrethroids including DEL and BT. The ethical clearance was approved by the Ethical Review Board at the Faculty of Public Health, Mahidol University.

#### 2.1.3. Study Area and Study Site

**Figure 4:**
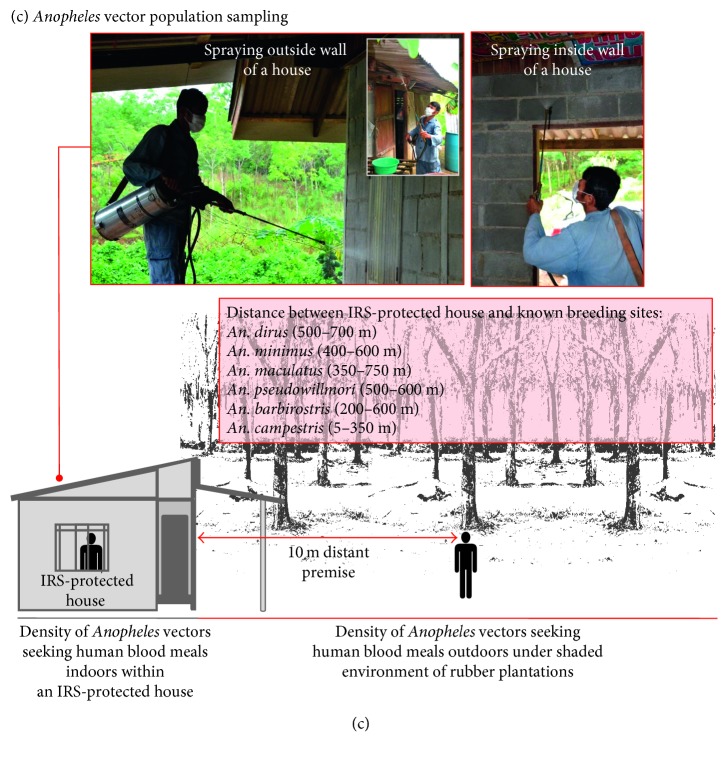
Study area and study site. (a) MRP ecotope at Khlong Khak village, Bo Rai district, Trat Province, elicited a malaria outbreak in 2013-2014. All malarial infections were epidemiologically linked with the indigenous *Plasmodium* infections in four separate MRP polygons within a 1.5 km^2^ MRP ecotope infested with *Anopheles* vectors. Data were modified from Kaewwaen and Bhumiratana [6]. (b) As similar to the other *Anopheles* sampling site I, the *Anopheles* sampling site II reflected households with rubber plantation practices whose members acquired the *Plasmodium* infections through mosquito-borne transmission. This MRP ecotope had strong density of *Anopheles* larval breeding sites. It was used in periodic assessment of species composition, abundance, and blood-feeding behaviors of *Anopheles* vectors at a house of rubber plantation polygon TBDKK3C as a study site. (c) A IRS-protected house of TBDKK3C polygon was used to collect *Anopheles* vector population samples by human landing catches. The distance of this IRS-protected house was estimated using the nearest certain *Anopheles* larval breeding sites within Khlong Khak MRP ecotope. The IRS using BT was described in detail in the text.

The Khlong Khak MRP ecotope covers a 1.5 km^2^ *Anopheles*-infested land area of rubber plantations. It is located on the latitude and longitude coordinates, 12°29′43.1″N to 12°30′15.6″N and 102°36′56.8″E to 102°37′45.9″E, which correspond to the Universal Transverse Mercator (UTM) coordinate system as shown in Figure 4. This MRP ecotope established malaria infection pockets in four separate rubber plantation polygons (Figures 4 and 4). In 2013, the introduced infections with *P. vivax* affected two rubber tappers living in the same rubber plantation polygon, TBDKK3A, and other accompanying person in the neighbor polygon, TBDKK3D. The infection with *P. falciparum* affected two rubber tappers living in two neighbor polygons, TBDKK3B and TBDKK3C. In 2014, the infection with *P. malariae* affected one more rubber tapper living in TBDKK3C.

Prior to the study, *Anopheles* larval surveys were carried out, based on the initial assessment of potential density of *Anopheles* larval breeding sites within Khlong Khak MRP ecotope, in assistance of the entomological survey teams of the VBDU 6.4.4 Bo Rai and the VBDC 6.4 Trat. A TBDKK3C polygon was then selected as the study site for the *Anopheles* sampling site II (Figure 4). This site met the selection criteria including the nearest larval breeding sites of some known primary vectors including *An. dirus*, *An. maculatus*, and *An. minimus*, the combination of human-vector interactions through the night time rubber plantation practices based on the vulnerability that two exposed rubber tappers acquired the *P. falciparum* and *P. malariae* infections through frequent bites of some potential *Anopheles* vectors, and the likelihood of variations of blood-seeking behaviors and flight ranges in local *Anopheles* vectors (i.e., primary, secondary, and suspected vectors). This selection permitted a house of this TBDKK3C polygon, which was treated by IRS using the BT insecticide in May 2015 (Figure 4(c)). Then, the IRS-protected house was used to monitor *Anopheles* adult vector populations sampled by human landing catches. The insecticide susceptibility of pooled population samples of some potent *Anopheles* vectors was tested against the pyrethroids (both DEL and BT) that had been used in IRS for vector control in the study area. Protocols for the mosquito collection and species identification of *Anopheles* adult vector population as well as insecticide susceptibility were described later. In addition, the population samples of locally adapted *Anopheles* vectors for this sampling site II in the Khlong Khak MRP ecotope were compared to those collected from the other *Anopheles* sampling site I. This site I was used as a sentinel site for baseline entomological surveillance for malaria to assess periodically species composition and abundance of *Anopheles* vector populations in malaria transmission control areas as part of the NMCP (Figure 4).

### 2.2. Indoor Residual Spray (IRS) Using Bifenthrin

The IRS currently used in this study was guided by World Health Organization (WHO) [14, 15]. Briefly, the insecticide was formulated as water-dispersible powder (WP) of BT 10% w/w as an active ingredient. A 50 gram powder of BT, that is, Bistar 10 WP prepacked by Chemfleet Co., Ltd., Bangkok, was made only for use in vector control implemented by the Thai NMCP. The insecticide was freshly prepared by suspending it well into 7.5 L of rain water in a plastic bucket under endemic conditions. By using a hand-compression sprayer, the suspension was then added into the stainless steel tank with a tank cover. Approximately 50–55 full strokes, which corresponded to 50–55 psi as measured by a pressure gauge, were applied to discharge the insecticide onto the walls, windows, doors, and ceilings of the house both inside and outside (Figure 4(c)). The active ingredient of BT was at a concentration of 25 mg/m^2^, as recommended by WHO [14].

### 2.3. Assessment of IRS Effects on the Adaptation of Local *Anopheles* Vector Populations

#### 2.3.1. Periodic Assessment of *Anopheles* Population Dynamics

In order to assess the effects of IRS on the adaptation of local *Anopheles* vector populations, the periodic assessment of *Anopheles* species composition, abundance, and blood-seeking behaviors was conducted using the IRS-protected house under agrienvironmental climatic conditions of Khlong Khak MRP ecotope as mentioned above. The indoor and outdoor collections of *Anopheles* mosquito populations were done in pre-IRS, during IRS, and post-IRS. In pre-IRS, collections were done in the early May 2015. During IRS, collections were done in the late May 2015. In post-IRS, collections were done in August 2015 (after 3 months), November 2015 (after 6 months), and May 2016 (after 12 months) (Figure 3).

#### 2.3.2. Human Landing Catch Collection and Species Identification

Human landing catch collection of blood-feeding *Anopheles* mosquitoes [16] was carried out between May 2015 and May 2016. A scheme for sampling *Anopheles* vector populations was designed by the following parameters: (i) the duration at which primary vectors, *An. dirus*, *An. maculatus*, and *An. minimus*, and their counterparts seek blood meals [5, 6, 10, 11], (ii) the duration at which the potency of IRS using BT lasted for as long as 6 months, (iii) the rainy-to-winter season during which abundance and distribution of *Anopheles* mosquitoes was upregulated, and (iv) the season during which the harvest of natural rubber was performed by rubber tappers [5, 6].

As to collect both endophagous and exophagous *Anopheles* vectors that sought human blood meals, the outdoor collection was performed within a premise of 10-meter distance from the indoor collection. At the time of collection, the *Anopheles* population samples were caught by four collectors using the aspirators. The indoor and outdoor collections were performed with the nocturnal periodicity for three consecutive days between 18:00 h and 06:00 h on the next morning. In every hour, a 45-minute landing catch was done followed by a 15-minute cessation. Also, temperature and humidity were recorded. All wild-caught *Anopheles* mosquitoes per hour were placed into the plastic cups covered with nylon, transferred to the field-based station, and then kept under a suitable condition of feeding a 10% glucose solution. For the identification of *Anopheles* species [17], the adult female mosquitoes individually transferred into new glass tubes were morphologically examined under a stereomicroscope.

### 2.4. Insecticide Susceptibility Test

The insecticide susceptibility test of using the pooled population samples of some potent *Anopheles* vectors was performed according to the conventional method described by WHO [18, 19]. Primary vectors, *An. dirus* and *An. minimus*, and their counterpart species, *An. campestris*, were likely to elicit seasonal abundance. However, the sample populations of only two predominantly abundant *Anopheles* vectors including *An. dirus* and *An. campestris* were subjected to the susceptibility test against the pyrethroids currently used in the IRS in the study MRP ecotope as described below. *An. minimus* whose female population numbers were very small at each time of collection was excluded.

Briefly, the pooled population samples of *An. dirus* and *An. campestris* female adults that were obtained at each time of collection were placed into each respective holding tube configuration of the test and control. In the test experiment, the holding tube contained a paper either treated with 0.05% DEL or with 0.09% BT. The concentration of pyrethroid was recommended as diagnostic single dose [18]. The sample population(s) of *An. dirus* and *An. campestris* female adults was separately exposed to the pyrethroid for one hour. In the control experiment, the female population(s) was exposed to untreated paper for the same one hour. Immediately after exposure, the exposed female mosquitoes were transferred to clean holding tube(s), and cotton pads soaked with 10% glucose solution were provided. The number of knockdown or dying mosquitoes was recorded within 24 h postexposure. Similarly, the nonexposed female adult mosquitoes were treated as before, and the number of dying or dead female adult mosquitoes was also recorded. If any, the replicates of paired tests and controls for each insecticide were performed. Seventy-nine percent (62/79) of the *An. dirus* female adults tested against the pyrethroids were collected at pre-IRS (42/55) and during IRS (20/24). Eighty-one percent (290/359) of *An. campestris* female adults that were collected at 3-month (65/68) and 6-month (225/291) post-IRS were used.

### 2.5. Data Analysis

The principal outcomes of this study were analyzed to explore *Anopheles* species composition, abundance, and blood-feeding behaviors and to see whether the IRS using BT currently available for the vector control could reduce the density of endophagous versus exophagous *Anopheles* vector populations. Descriptive statistics were used to present the number and percent of night-biting *Anopheles* vector populations that were collected in pre-IRS, during IRS, and post-IRS at 3, 6, and 12 months. Given its fluctuation constrained by seasonal variations, the *Anopheles* vector abundance only constrained by the effects of IRS was observed. Thus, the significant difference in the abundance (the mean density or human landing rate) of three predominant night-biters *An. dirus*, *An. minimus*, and *An. campestris* periodically monitored by indoor and outdoor collections was tested using the one-way ANOVA for two independent samples (*P* < 0.05).

To assess the susceptibility of *An. dirus* and *An. campestris* to the pyrethroids, both DEL and BT, currently available for use in vector control in the study MRP ecotope, the mortality of *Anopheles* vector populations in paired tests and controls was analyzed. The mortality (%) of exposed populations of each test is mathematically expressed as observed mortality which is equal to total number of dead/dying populations divided by total sample size and multiplied by 100. Similarly, the calculation for the control sample was made to obtain a value for the control mortality. The Abbots formula was used for the adjusted mortality, when the control mortality is greater than 5% but less than 20%, as follows: (% observed mortality–% control mortality)/(100–% control mortality) × 100. If any, an estimate of the 95% confidence intervals was used for observed mortality in the test. The susceptibility of *An. dirus* and *An. campestris* to the pyrethroids was qualitatively assessed as high (98–100% mortality), moderate (80–97% mortality), and low (<80% mortality).

## 3. Results

### 3.1. *Anopheles* Species Composition, Abundance, and Blood-Feeding Behaviors

**Figure 5:**
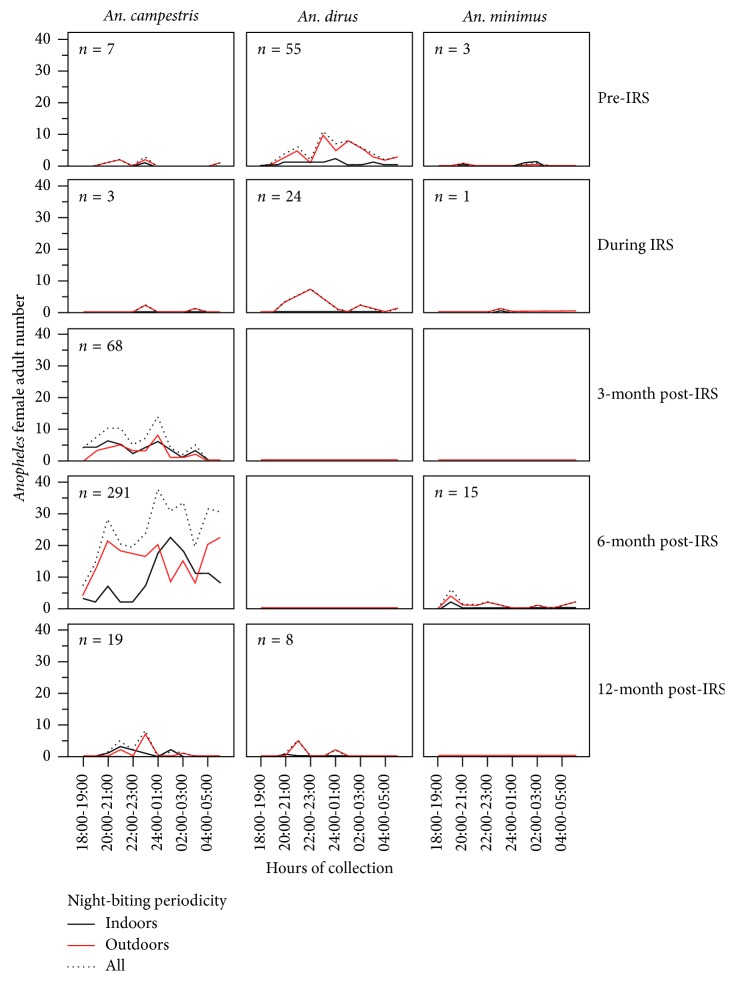
Comparison of the abundances of night-biting *An. dirus*, *An. minimus*, and *An. campestris* vectors in pre-IRS, during IRS, and post-IRS. The female adult populations of three predominantly night-biting *An. dirus*, *An. minimus*, and *An. campestris* vectors were compared using human landing catch collections. At the time of indoor and outdoor collections, night-biting periodicity is also shown for pre-IRS, during IRS, and post-IRS (i.e., 3-, 6-, and 12-month post-IRS).

**Figure 6:**
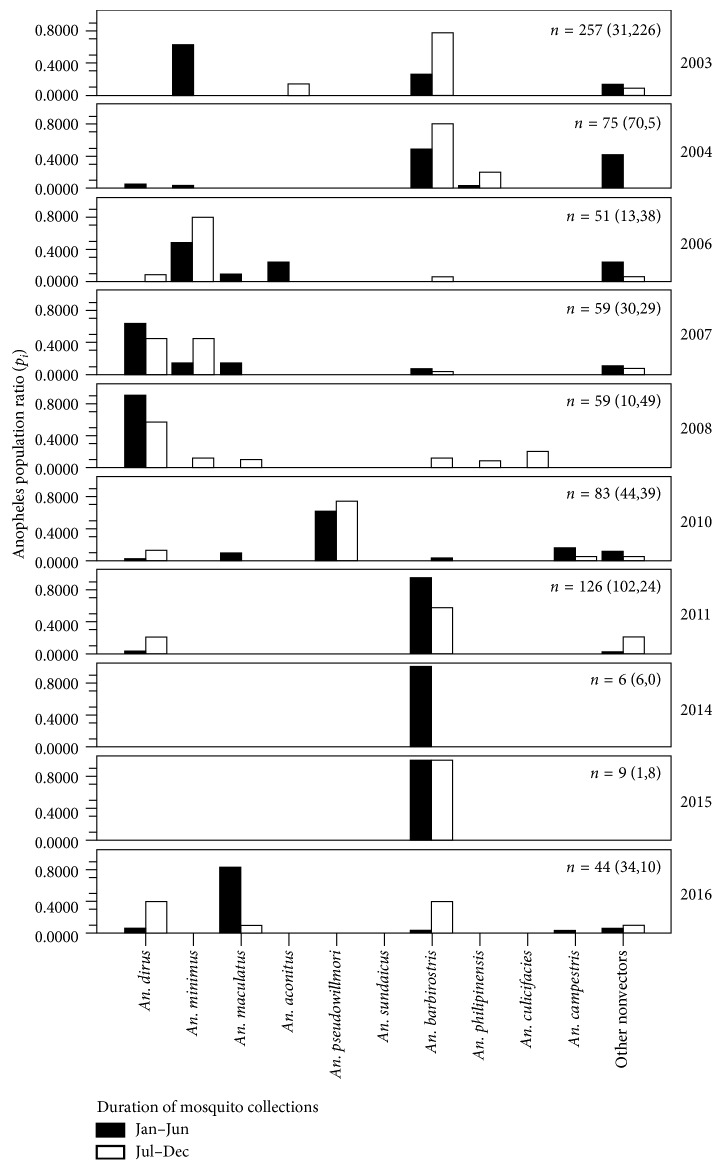
*Anopheles* populations adapted to the sampling site I based on baseline entomological surveillance data of *Anopheles* vector populations from the *Anopheles* sampling site I from 2003 to 2016. Variations of species composition and abundance are shown. The total number and the numbers (in parenthesis) collected between the first-half (front number, January to June) and the second-half (rare number, July to December) of each year are presented. The *Anopheles* population ratios (*p*_*i*_) were expressed by the number of individual species divided by the total number of all species observed at time of collection. Data were modified from the VBDC 6.4 Trat.

The study MRP ecotope demonstrated that it was likely to show the population dynamics (the species and number) of local *Anopheles* vectors with the nocturnal periodicity in the absence or presence of IRS (Tables 1 and 2 and Figure 5). Such findings compared well with findings of those indigenous to the *Anopheles* sampling site I (Figure 6). Based on human landing catch collections, a total of 519 *Anopheles* mosquitoes (population ratio or *p*_*i*_) included five species of malaria vectors: *An. campestris* (388; *p*_*i*_ = 0.747), *An. dirus* (87; *p*_*i*_ = 0.168), *An. minimus* (19; *p*_*i*_ = 0.037), *An. barbirostris* (14; *p*_*i*_ = 0.027), and *An. pseudowillmori* (1; *p*_*i*_ = 0.002), respectively. *An. jamesii* (totally 10; *p*_*i*_ = 0.019) was the only one species of nonvector found throughout the study.

Particularly, three predominantly anthropophagous vectors, namely, *An. dirus*, *An. minimus*, and *An. campestris*, were likely to show night-biting preference with two peaks during the night time regardless of the IRS effects (Figure 5). *An. dirus* had the first peak between 21:00 and 23:00 h and the second peak between 24:00 and 02:00 h. Similarly, *An. minimus* had the first peak between 19:00 and 21:00 h and the second peak between 01:00 and 03:00 h. As compared to *An. dirus* and *An. minimus* counterparts, *An. campestris* started to seek blood meal from 19:00 to 06:00 h but the first peak before midnight, starting from 20:00 to 22:00 h, and after midnight occurring between 01:00 and 03:00 h. Moreover, these three abundant *Anopheles* vectors were more likely to behave exophagy than endophagy (Tables 1 and 2 and Figure 5). For instance, there were more exophagous versus endophagous vector populations: *An. campestris* (230 versus 158), *An. dirus* (78 versus 9), and *An. minimus* (16 versus 3).

### 3.2. IRS Effects on the Abundance of *An. dirus*, *An. minimus*, and *An. campestris*

**Figure 7:**
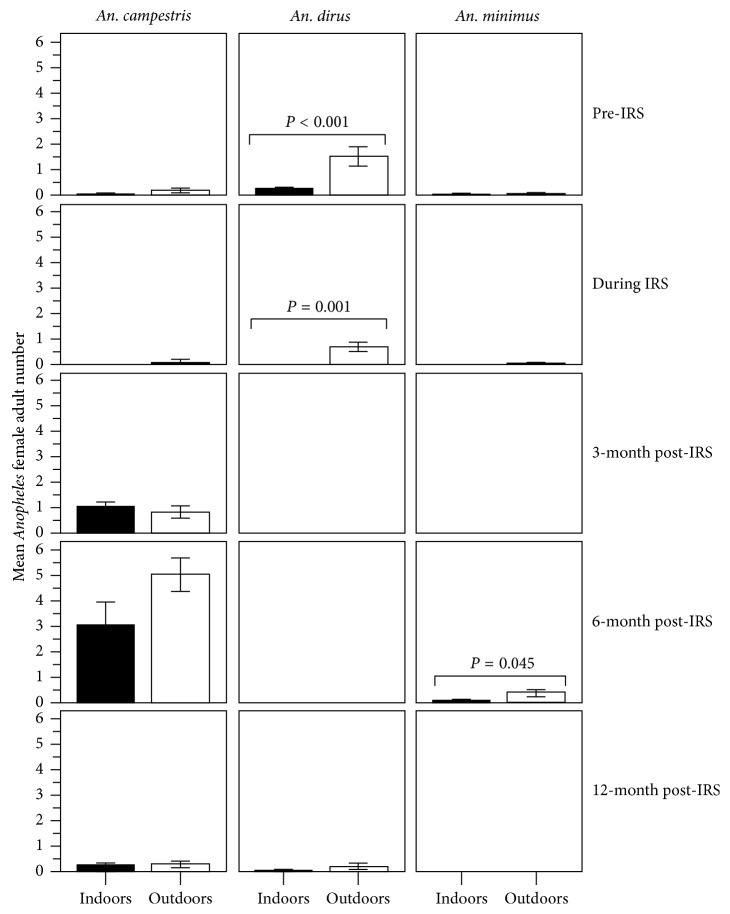
IRS effects on the abundances (mean densities) of night-biting *An. dirus*, *An. minimus*, and *An. campestris* vectors in pre-IRS, during IRS, and post-IRS. Effects of IRS on the abundance of three predominantly night-biting *An. dirus*, *An. minimus*, and *An. campestris* vectors were compared between indoor and outdoor densities (mean female adult numbers) of *Anopheles* vectors at pre-IRS, during IRS, and post-IRS (i.e., 3-, 6-, and 12-month post-IRS). The significance at *P* < 0.05 is shown.

**Figure 8:**
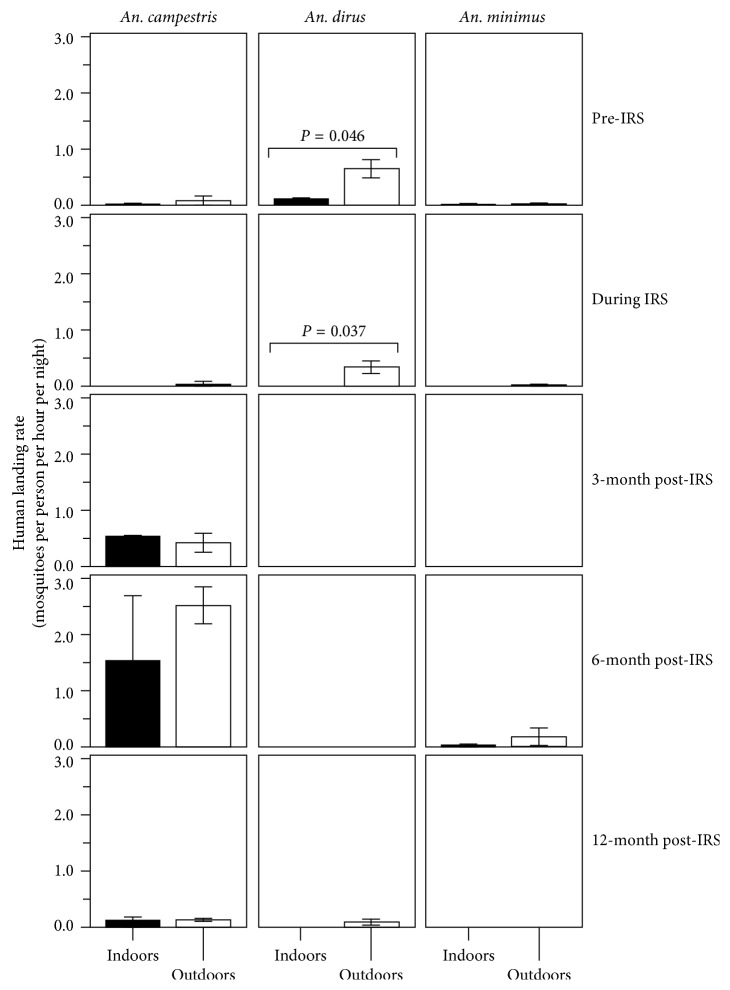
IRS effects on the abundances (human landing rates) of night-biting *An. dirus*, *An. minimus*, and *An. campestris* vectors in pre-IRS, during IRS, and post-IRS. Similar to Figure 7, IRS effects were observed on the human landing rates of three predominantly night-biting *An. dirus*, *An. minimus*, and *An. campestris* vectors by comparing indoor and outdoor collections of *Anopheles* vectors at pre-IRS, during IRS, and post-IRS (i.e., 3-, 6-, and 12-month post-IRS). The significance at *P* < 0.05 is shown.

Provided that IRS had the effects on density reduction of *Anopheles* vectors seeking blood meals in the IRS-protected house, the abundances of three predominant night-biters *An. dirus*, *An. minimus*, and *An. campestris*, which were obtained by indoor and outdoor collections, were compared at different time periods in pre-IRS, during IRS, and post-IRS. The *Anopheles* vector abundance relied on two entomological indices, mean density (Figure 7) and human landing rate (female mosquitoes per person per hour per night) (Figure 8).

Obviously, the IRS had the effects on the abundance of these responsible *Anopheles* vectors that behaved endophagously. There was a significant difference in the abundance of exophagous versus endophagous population samples of *An. dirus* observed at pre-IRS (*P* < 0.001 for mean density; *P*=0.046 for human landing rate) and during IRS (*P*=0.001 for mean density; *P*=0.037 for human landing rate) (Figures 7 and 8 and Table S1). The abundance of *An. dirus* and *An. minimus* that fed indoors was likely to be influenced by the IRS. Obviously, *An. dirus* disappeared at 3-month and 6-month post-IRS but reappeared at 12-month post-IRS. *An. minimus* disappeared during IRS and at 3-month and 12-month post-IRS but reappeared at 6-month post-IRS. There was a significant difference in the mean density of exophagous versus endophagous population samples observed at 6-month post-IRS (*P*=0.045) (Figure 7 and Table S1).

By contrast, *An. campestris* had a tendency to feed outdoors and indoors, while appearing over a period of the study in the presence of IRS (Figures 7 and 8 and Table S1). *An. campestris* had a tendency to increase more abundance, that is, increasing mean density or human landing rate at 6-month post-IRS rather than that observed at 3-month and 12-month post-IRS. However, there was no significant difference in the abundance (neither mean density nor human landing rate) of exophagous versus endophagous *An. campestris* population samples observed in pre-IRS, during IRS, and post-IRS, whether 3, 6, or 12 months.

### 3.3. Susceptibility of *An. campestris* and *An. dirus* to the Pyrethroids

Only the pooled population samples of *An. campestris* and *An. dirus* were used in testing their susceptibility to the pyrethroids, both DEL and BT, in this study. *An. campestris* had a tendency to be moderately susceptible to 0.05% DEL and 0.09% BT, showing the observed mortality rates (95% CI) of 87.2% (78.4–95.9) and 95.0% (79.1–100) (Table 3). *An. dirus* had a tendency to be highly susceptible to 0.05% DEL and 0.09% BT, showing the same 100% mortality rate (Table 3). Additionally, *An. dirus* population samples that were collected by human landing catches from other *Anopheles* sampling sites of the Bo Rai district were also highly susceptible to 0.05% DEL with 100% mortality rate.

## 4. Discussion

The study MRP ecotope contributed greatly to favor the adaptation of local *Anopheles* vectors that can infest rubber plantations as receptive environment of the recent 2013-2014 malaria outbreak. Our findings compared well with findings obtained from the *Anopheles* sampling site I that this MRP ecotope created agrienvironmental climatic conditions suitable for the diversity of *Anopheles* vectors. This driver may be associated with the emergence of local malaria transmission dynamics caused by *Plasmodium* malarial parasites in Khlong Khak MRP ecotope. The present study demonstrated that, if expected to accompany changes in their responses to the insecticides currently used in the vector control as part of the NMCP, the variation in species composition, abundance, and blood-feeding behaviors of local *Anopheles* vectors was deemed necessary for surveillance and monitoring of residual malaria parasite transmission in *Anopheles* vectors. The adaptation of primary and suspected *Anopheles* vectors sessile to receptive environment of Khlong Khak MRP ecotope was discussed in detail.

### 4.1. Primary *Anopheles* Vectors and IRS Effects

The *Anopheles*-infested land area of Khlong Khak MRP ecotope is covered by the rubber trees with dense canopies connecting to the hill. This MRP ecotope generates microclimatic conditions and shaded environment that a plethora of *Anopheles* vectors can infest or reinfest. The primary vectors adapted to local environment of the study MRP ecotope included *An. dirus*, *An. minimus*, and *An. maculatus*; all of which have their sibling species and exhibit abilities to transmit *P. falciparum* and *P. vivax* malaria [20–24]. We found that *An. dirus* requires particular environment for breeding larvae in standing water or low-flowing water of small, shallow rock pools with mud/soil/sand and plant debris. These shaded rock pools are located on the hillside or foothills, on the altitudes between 70 and 120 meters above sea level (MASL). Both *An. dirus* and *An. maculatus* cobred in some shaded rock pools, while *An. minimus* bred in a stream of low-moving water with vegetation on the altitudes between 30 and 50 MASL. Given its seasonal abundance, human blood-feeding behaviors, and flight ranges, *An. dirus* and *An. minimus* were predominant night-biters in the study MRP ecotope. Meanwhile, *An. maculatus* seemed not to seek any human blood meal despite the fact that there existed more available breeding sites, and further investigation is required.

Such primary vectors resting outdoors may have abilities to seek human blood meals within their flight ranges. We found that both *An. dirus* and *An. minimus* night-biters had a tendency to feed outdoors rather than indoors in the absence or presence of the IRS using BT. Apart from the dynamics of *Anopheles* vector populations, that is, constrained seasonally and geographically by ecological relationship in nature, the fluctuation of their abundance and feeding habits observed in this study could be influenced by the IRS. Our findings compared well with previous findings that as compared to *An. minimus* [22, 23] and *An. maculatus* [20], *An. dirus* was likely to behave both exophagy and endophagy, but significantly, it was a exophilic anthropophagous feeder [21]. With respect to their blood-feeding behaviors that responded to the IRS, these primary vectors seemed to feed outdoors rather than indoors, showing mean densities or human landing rates for outdoor feeding greater than that for indoor feeding in pre-IRS, during IRS, and post-IRS. The findings suggested that blood-feeding behaviors of both *An. dirus* and *An. minimus* were likely to avoid feeding indoors in the IRS-protected house [25, 26]. On the contrary, the IRS using BT that had the potency for as long as 6 months might have the effects on reducing indoor densities of these primary vectors [25–27], particularly endophilic anthropophagous *An. dirus* night-biter during IRS and post-IRS. We do not have additional data that the IRS using BT or DEL has knockdown effects or excite-repellency effects. However, *An. dirus* was more sensitive to the IRS using BT than *An. minimus*. Due to its potency, the effects of IRS might greatly reduce the abundance of *An. dirus* within 6 months of post-IRS, but not *An. minimus* significantly feeding outdoors. The IRS might be slightly effective at 12-month post-IRS against *An. dirus*. Based on the insecticide susceptibility testing, *An. dirus* had a tendency to be highly susceptible (100% mortality) to both DEL and BT currently used in the IRS. As compared to BT, DEL exerted the knockdown effects that could kill rapidly the sample populations when contacted within one hour. These observations implied that tested population samples of the local *An. dirus* vector, as well as *An. minimus*, had a tendency to be highly sensitive to the pyrethroids tested in this study.

Such findings implied that if expected to accompany changes in their behaviors, both *An. dirus* and *An. minimus* can pose the risk for residual malaria parasite transmission in these vectors in the study MRP ecotope due likely to the decreased potency of IRS. Further investigation is however required to better understand whether changes in behavioral responses to the IRS using BT or other pyrethroids are consistent with the genetic and physiochemical constraints [25–27]. If there are limits of the insecticides used in the IRS, any vulnerable person might not be sheltered by the IRS because such night-time rubber tapping might render them frequently exposed to outdoor biting of those primary vectors. If expected to accompany risk health behaviors, they would be at the greater risks for the infections as similar to those infected with *Plasmodium* infections in this study MRP ecotope.

### 4.2. Suspected *Anopheles* Vectors and IRS Effects

Based on their abundance and distribution, other potentially suspected vectors adapted to the local environment of rubber plantations in the study MRP ecotope included *An. campestris* and *An. barbirostris* because they were likely to potentially transmit *P. vivax* in Thailand [28–30]. Recently in Cambodia, Durnez et al. [31] demonstrated that, based on cow-baited tent collection, *An. barbirostris* was shown for a natural host of carrying *P. falciparum* parasite. Among these vectors that rested outdoors and likely behaved zoophagy, *An. campestris* had a tendency to play role in transmission of *P. vivax* in this MRP ecotope rather than its counterparts, *An. barbirostris* and *An. pseudowillmori*, due likely to its abundance and distribution. Obviously in the study MRP ecotope, *An. campestris* could breed in man-made pools with vegetation, shallow wells, and in standing or low-flowing water of small, shallow mud/soil pools with some plant debris on the altitude of approximately 30 MASL. The availability of these larval habitats was commonly seen in this MRP ecotope all the year round. Particularly, both larvae and adult population numbers increased with increasing rainfall. This is a reason why *An. campestris* as most abundant species had more advantageous competition in the absence or presence of the IRS in the study MRP ecotope.

More interestingly, *An. campestris* had a tendency to bite indoors in the IRS-protected house. We argued that, in comparison with those observed at pre-IRS and during IRS, there existed elevated numbers of local *An. campestris*, especially observed at 3- and 6-month IRS. The observed endophagous numbers of *An. campestris* suggested the forces, but not the selection, exerted by the tolerability to the insecticides, both DEL and BT, currently used in the IRS in this MRP ecotope. The local *An. campestris* populations had a tendency to be moderately susceptible (less than 98% mortality) to BT, but the species was likely to be resistant to the DEL tested in this study. On the contrary, the IRS using these insecticides did not reduce the indoor densities of *An. campestris*, hence rendering any vulnerable persons more likely susceptible to the *P. vivax* infection although sheltered by the IRS. If there are limits of the potency of DEL or BT used in the IRS, further investigation will be needed to better address the use and coverage of the IRS using these pyrethroids whether it could reduce human-vector contact in the IRS-protected house through the acquisition to bites of *An. campestris* as compared to that of *An. dirus* and *An. minimus*.

## 5. Conclusion and Suggestions

The study MRP ecotope was geographically associated with the infestation or reinfestation of local *Anopheles* vectors sessile to receptive environment of rubber plantations. The agrienviornmental climatic conditions of the MRP ecotope may influence the dynamics of *Anopheles* vector populations based radically on variations of species composition, abundance, and blood-feeding behaviors. Three predominant *Anopheles* vectors including primary vectors (*An. dirus* and *An. minimus*) and suspected vector (*An. campestris*) might play roles in local malaria transmission dynamics in the study MRP ecotope. Also, all these target vectors are needed for surveillance and monitoring of residual malaria parasite transmission in the absence or presence of the implementation of core interventions of the vector control [32, 33] due likely to their seasonal abundance, outdoor biting preference, and tolerability to DEL and BT currently used in the IRS. In particular, if there exist any changes in behaviors for survival of local *Anopheles* vectors to the currently used pyrethroids, further study would better address the dynamics of local *Anopheles* vector populations adapted to physiochemical environment of the MRP ecotope and insecticide susceptibility of responsible *Anopheles* vectors that serve as key determinants of malaria risks [25–27]. This would reflect effective vector surveillance and control of residual malaria parasite transmission in receptive environment such as rubber plantations, in addition to what are recommended by WHO [13, 34].

## Figures and Tables

**Table 1:** Periodic assessment of endophagous *Anopheles* species composition and abundance by night-time human landing catches in pre-IRS, during IRS, and post-IRS.

Species	Status	Number (%) of indoor-biting *Anopheles* mosquitoes				
		Pre-IRS	During IRS	3-month post-IRS	6-month post-IRS	12-month post-IRS
*An. dirus*	Primary	8 (53.3)	0	0	0	1 (10.0)
*An. minimus*	Primary	1 (6.7)	0	0	2 (1.8)	0
*An. barbirostris*	Suspected	2 (13.3)	3 (100.0)	0	0	0
*An. campestris*	Suspected	1 (6.7)	0	38 (100)	110 (98.2)	9 (90.0)
*An. jamesii*	Nonvector	3 (20.0)	0	0	0	0
Total		15	3	38	112	10

**Table 2:** Periodic assessment of exophagous *Anopheles* species composition and abundance by night-time human landing catches in pre-IRS, during IRS, and post-IRS.

Species	Status	Number (%) of outdoor-biting *Anopheles* mosquitoes				
		Pre-IRS	During IRS	3-month post-IRS	6-month post-IRS	12-month post-IRS
*An. dirus*	Primary	47 (72.3)	24 (72.7)	0	0	7 (38.9)
*An. minimus*	Primary	2 (3.1)	1 (3.0)	0	13 (6.7)	0
*An. pseudowillmori*	Secondary	1 (1.5)	0	0	0	0
*An. barbirostris*	Suspected	4 (6.2)	5 (15.2)	0	0	0
*An. campestris*	Suspected	6 (9.2)	3 (9.1)	30 (100)	181 (92.8)	10 (55.6)
*An. jamesii*	Non vector	5 (7.7)	0	0	1 (0.5)	1 (5.5)
Total		65	33	30	195	18

**Table 3:** Susceptibility of *An. campestris* and *An. dirus* to deltamethrin and bifenthrin.

	Total number of samples		Mortality (%)		95% CI for observed mortality in test
	Test	Control	Test	Control	
*An. campestris* ^b^					
Deltamethrin, 0.05%	140	32	87.2	0.0	78.4–95.9
Bifenthrin, 0.09%	88	30	95.0^d^	13.3	79.1–100.0

*An. dirus* ^b^					
Deltamethrin, 0.05%	32	10	100.0	0.0	NA
Bifenthrin, 0.09%	10	10	100.0	0.0	NA

*An. dirus* ^c^					
Deltamethrin, 0.05%	60	10	100.0	0.0	NA

NA: not applicable. ^a^Population samples obtained at 3- and 6-month post-IRS from the study MRP ecotope. Population samples obtained between pre-IRS and during IRS from the study MRP ecotope as the *Anopheles* sampling site II^b^, and additionally, other *Anopheles* sampling sites of Bo Rai district^c^. ^d^Abbot's formula applied to correct observed mortality in the test.

## Data Availability

The data used to support the findings of this study are available from the corresponding author upon request.

## Abbreviations

ANOVA: Analysis of variance
BT: Bifenthrin
DEL: Deltamethrin
IRS: Indoor residual spraying
ITNs: Insecticide-treated nets
LLINs: Long-lasting insecticidal nets
MRP: Malaria-associated rubber plantations
NMCP: National malaria control program
ODPC: Office of disease prevention and control
UTM: Universal transverse mercator
VBDC: Vector-borne disease control center
WHO: World Health Organization.

## Supplementary Materials

Supplementary Materials Table S1: effects of IRS on the abundances (outdoor versus indoor densities) of night-biting *An. dirus*, *An. minimus*, and *An. campestris*.
