# Supplementary material for: Adaptation of Anopheles Vectors to Anthropogenic Malaria-Associated Rubber Plantations and Indoor Residual Spraying: Establishing Population Dynamics and Insecticide Susceptibility
Source: Can J Infect Dis Med Microbiol. 2018 Jun 21;2018:9853409. doi: 10.1155/2018/9853409 (PMC6032653; doi:10.1155/2018/9853409)
Supplement: Supplementary Materials — Table S1: effects of IRS on the abundances (outdoor versus indoor densities) of night-biting An. dirus, An. minimus, and An. campestris. [file 9853409.f1.docx]

**Table S1** Effects of IRS on the abundances (outdoor vs. indoor densities) of night-biting *An. dirus*, *An. minimus*, and *An. campestris*

|  | Abundance (mean±SD) | |  |
| --- | --- | --- | --- |
|  | Outdoors | Indoors | *P*-value |
| ***An. dirus*** |  |  |  |
| Pre-IRS | 1.31±1.64 | 0.22±0.42 | F = 14.81, *P* < 0.001^*^ |
| During IRS | 0.67±1.12 | 0.0 | F = 12.73, *P* = 0.001^*^ |
| 3-month post IRS | 0.0 | 0.0 | NA |
| 6-month post IRS | 0.0 | 0.0 | NA |
| 12-month post IRS | 0.19±0.75 | 0.03±0.17 | F = 1.70, *P* = 0.197 |
| ***An. minimus*** |  |  |  |
| Pre-IRS | 0.06±0.23 | 0.03±0.17 | F = 0.340, *P* = 0.562 |
| During IRS | 0.03±0.17 | 0.0 | F = 1.000, *P* = 0.321 |
| 3-month post IRS | 0.0 | 0.0 | NA |
| 6-month post IRS | 0.36±0.83 | 0.06±0.33 | F = 4.172, *P* = 0.045^*^ |
| 12-month post IRS | 0.0 | 0.0 | NA |
| ***An. campestris*** |  |  |  |
| Pre-IRS | 0.16±0.50 | 0.03±0.17 | F = 2.340, *P* = 0.131 |
| During IRS | 0.08±0.37 | 0.0 | F = 1.842, *P* = 0.179 |
| 3-month post IRS | 0.83±1.36 | 1.06±1.12 | F = 0.571, *P* = 0.452 |
| 6-month post IRS | 5.03±3.90 | 3.06±5.32 | F = 3.214, *P* = 0.077 |
| 12-month post IRS | 0.28±0.74 | 0.25±0.55 | F = 0.032, *P* = 0.858 |

^*^Statistically significant with One-Way ANOVA for two independent samples.
